# A triazine-based BODIPY trimer as a molecular viscometer†
†Electronic supplementary information (ESI) available: Synthesis of **1** and **2**, spectroscopic measurements, vesicle preparations, and additional spectral and imaging characterization. See DOI: 10.1039/c5cp07214j



**DOI:** 10.1039/c5cp07214j

**Published:** 2016-01-21

**Authors:** Sangram L. Raut, Joseph D. Kimball, Rafal Fudala, Ilkay Bora, Rahul Chib, Hana Jaafari, Marlius K. Castillo, Nicholas W. Smith, Ignacy Gryczynski, Sergei V. Dzyuba, Zygmunt Gryczynski

**Affiliations:** a Department of Physics and Astronomy , Texas Christian University , 2800 S. University Dr. , Fort Worth , TX 76129 , USA . Email: Z.gryczynski@tcu.edu ; Fax: +1 817 257 7742 ; Tel: +1 817 257 4209; b Department of Cell Biology and Immunology , University of North Texas Health Science Center , Fort Worth , TX 76129 , USA; c Department of Chemistry and Biochemistry , Texas Christian University , 2800 S. University Dr., Fort Worth , TX 76129 , USA . Email: s.dzyuba@tcu.edu ; Fax: +1 817 257 5851 ; Tel: +1 817 257 6218

## Abstract

Photophysical behaviour of a novel trimeric BODIPY rotor with a high extinction coefficient is reported. Steady state and time resolved fluorescence measurements established that the trimer could be used as a viscometer for molecular solvents, membrane-like environments and several cancer cell lines.

## Abstract 2

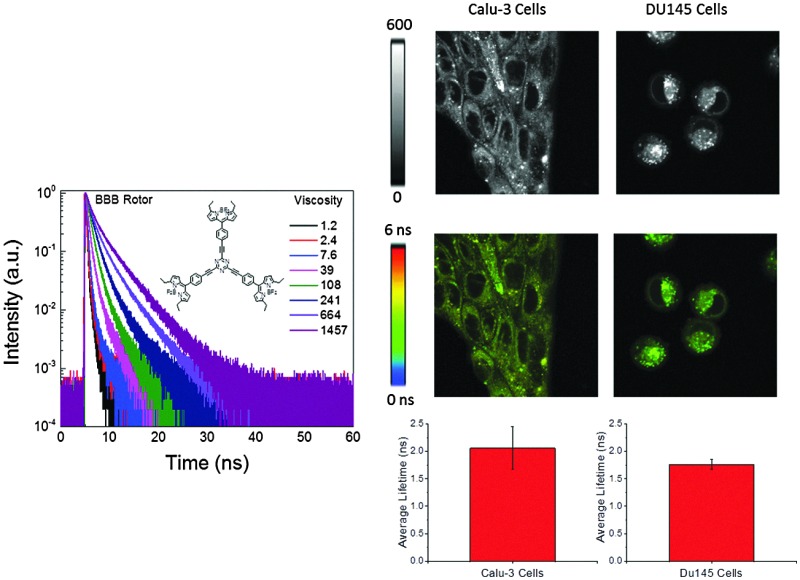
Photophysical behaviour of a novel trimeric BODIPY rotor with a high extinction coefficient is reported.

## Introduction

Viscosity is one of the fundamental physical properties of fluids, including biological and physiological components of tissues and cells. An ability to monitor the viscosity of the biological environments with high spatial resolution and to determine viscosity changes upon exposure to particular stimuli has immediate practical applications for biological, medical and materials studies.^1–5^ However, current capabilities of monitoring the viscosity on cellular and sub-cellular levels are limited. Primarily this stems from the fact that currently available probes have low molar extinction coefficients and they are often sensitive to other environmental parameters, such as polarity. In addition, from the synthetic point of view, access to molecular viscometers can require multistep syntheses. Thus, efforts that focus on developing technologies and probes that would be able to monitor the viscosity on a molecular level are important. Recently, there has been increased interest in developing fluorescent small molecular probes that could act as molecular viscometers in a variety of environments.^6,7^ Such probes exhibit photophysical properties (brightness, Stokes shifts, lifetimes, *etc.*) that can be tuned by synthetically modifying their structures. Various approaches^8,9^ as well different molecular rotors have been used to measure viscosity.^10–15^

Molecular viscometers that feature monomers (*i.e.*, single dye molecules with an auxiliary group) are primarily based on the fluorescent scaffold rotating around the single bound auxiliary segment, which upon excitation form a twisted state. Such a structural change occurs in the excited state and is accompanied by intramolecular charge transfer resulting in a strong modification of the fluorescence signal. From a practical point of view, molecular probes that have high extinction coefficients and that exhibit potentially high brightness would be desirable. BODIPY dyes, due to their unique and easily tunable photophysical properties, have been utilized for a variety of applications, including those related to viscosity. Specifically, Kuimova *et al.* and others have demonstrated the viability of BODIPY-based rotors decorated with alkyl, ether, ammonium, steroid and other types of moieties to act as molecular viscometers in various types of environments;^16,17–22^ an aldehyde containing BODIPY rotor was also shown to work as a viscosity sensor.^23^ Other types of single dye viscometers including SYBR Green and PicoGreen,^24^ thioflavin-T,^25^ and stilbene-based^26^ molecular rotors have also been reported. All these viscometers have molar absorption coefficients of less than 80 000 M^–1^ cm^–1^.

In addition, several homodimeric, small molecule viscometers have been described. For example, a porphyrin homodimer was reported to be used as an intracellular viscometer in apoptotic cells.^27,28^ A pyrene homodimer has also been recently utilized as a viscometer.^29^ A BODIPY–BODIPY rotor was shown to be a viable viscometer^30^ for various cellular, membrane-like environments, and its potential to be used for measuring the viscosity of molecular and ionic types of media was demonstrated.^14^ A viscosity sensitive ROFRET molecule featuring a BODIPY rotor connected to an alkylated BODIPY through a triazole-based spacer was disclosed.^31^ In addition, heterodimeric viscometers were explored. In particular, a heterodimeric Nile red-BODIPY dyad was reported to measure the micropolarity and microviscosity of endoplasmic reticulum in HeLa cells.^32^ A coumarin–BODIPY conjugate was found to be suitable for measuring the mitochondrial viscosity.^33^ In general, the aforementioned viscometers exhibited molar absorption coefficients of up to 150 000 M^–1^ cm^–1^ (*i.e.*, about twice the extinction coefficient of the monomeric type of rotors).

**Scheme 1:**
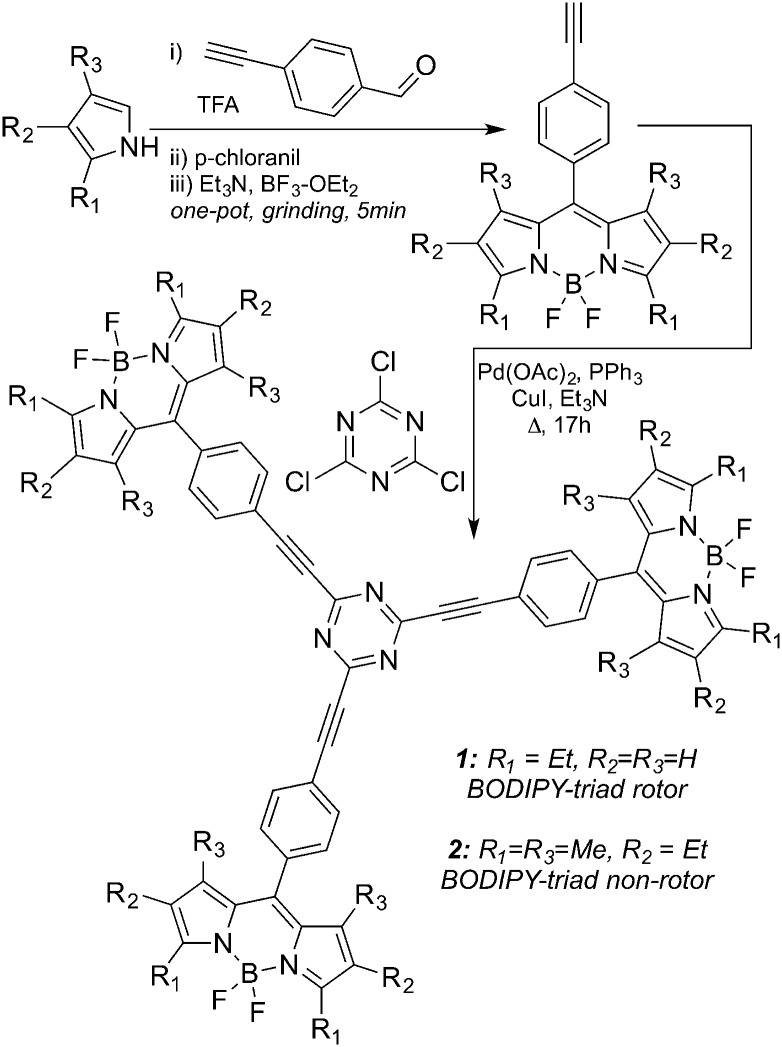
Synthesis of BODIPY trimers **1** and **2**.

Here, we report novel, easily accessible fluorescent trimeric BODIPY dyes **1** and **2** (Scheme 1) with molar absorption coefficients in the range of 200 000 M^–1^ cm^–1^ in a number of solvents (Table S1, ESI†). Dye **1** can act as a small molecule viscometer for molecular solvents as well as for cellular and membrane-like environments. Dye **2** has methyl substituents at 1 and 7 positions of the boron-dipyrromethene core that hinder the rotation of the substituents in the *meso*-position, thus limiting the range of the conformational changes. As a result, the change of the fluorescence signal as a function of media's viscosity was found to be relatively small and could be attributed to solvent properties unrelated to viscosity (*e.g.*, polarity, *etc.*).

## Results and discussion

1,3,5-Triazine is a convenient scaffold that allows assembling of multifunctional, structurally diverse systems.^34–39^ Incorporation of BODIPY dyes into the triazene core has also been reported^40^ and these types of systems were used as sensors (especially in metal ion recognition)^41,42^ as well as components of the dye-sensitized solar cells.^43,44^

In this study, we took advantage of the three reactive chlorine atoms of 2,4,6-trichloro-1,3,5-triazene (cyanuric chloride) to assemble three BODIPY dyes to synthesize BODIPY timers (Scheme 1 and ESI†). Specifically, alkyne-containing BODIPY dyes were prepared according to previously published procedures,^45,46^ and subsequent cross-coupling reactions furnished the BODIPY trimers **1** (rotor) and **2** (non-rotor) in just two steps from commercially available starting materials (Scheme 1).

### Photophysical characterization

The working principle of molecular viscometers is based on suppressing the non-radiative relaxation channel in a viscosity dependent manner.^47^ In short, the relative displacement/rotation of two chromophores in the excited state leads to intramolecular charge transfer and the concomitant quenching of fluorescence.^7,48,49^ These molecules exhibit two types of de-excitation pathways once they are in the excited state: a fluorescence radiative pathway and a non-radiative de-excitation pathway. The fluorescence characteristics of the probe depend on the freedom of conformational change. For systems, where the intramolecular conformational change depends on the viscosity of the local environment in the proximity of the molecule a linear dependence of the quantum yield (*Φ*) and/or fluorescence lifetime (*τ*) as a function of medium's viscosity should be observed. This viscosity dependence of lifetime, for example can be expressed using Förster–Hoffman theory, according to the following equation:

1 ln(*τ*) = *C*′ + *y* ln(*η*)

where *η* is the viscosity of the medium, *C*′ is the *y*-axis intercept and *y* is the slope of the line, which is 0.6 for the perfect rotor as predicted by the Förster–Hoffman theory^47^ and *τ* is the fluorescence lifetime of the probe. This equation holds true for the emission quantum yield as well. However, fluorescence lifetime is a much more reliable property than the quantum yield due to its insensitivity to local dye concentration.

**Fig. 1:**
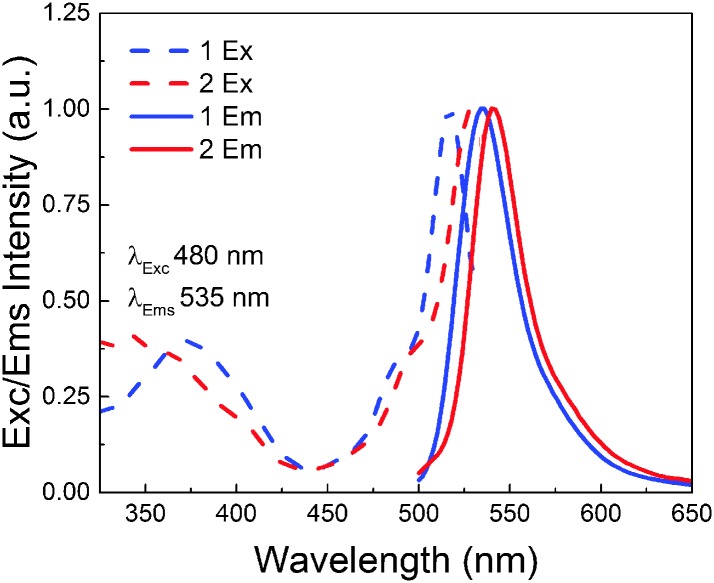
Excitation and emission spectra of **1** and **2**.

A small red shift in the excitation and emission spectra of rotor dye **1** compared to the ones of the non-rotor dye **2** in ethanol (**1**: *λ*_ex_ = 516 nm, *λ*_em_ = 535 nm; **2** = *λ*_ex_ = 528 nm, *λ*_em_ = 540 nm) was arguably attributed to the alkyl substitutions on the BODIPY core (Fig. 1). The methyl groups in the positions 1 and 7 on the BODIPY backbone prevent the rotation during excitation, thus making it insensitive to the changes of the surrounding viscosity. However, when these methyl groups are absent (which is the case of dye **1**) the rotation is possible which suppresses the non-radiative de-excitation processes, thus making the molecule sensitive to the surrounding viscosity.

**Fig. 2:**
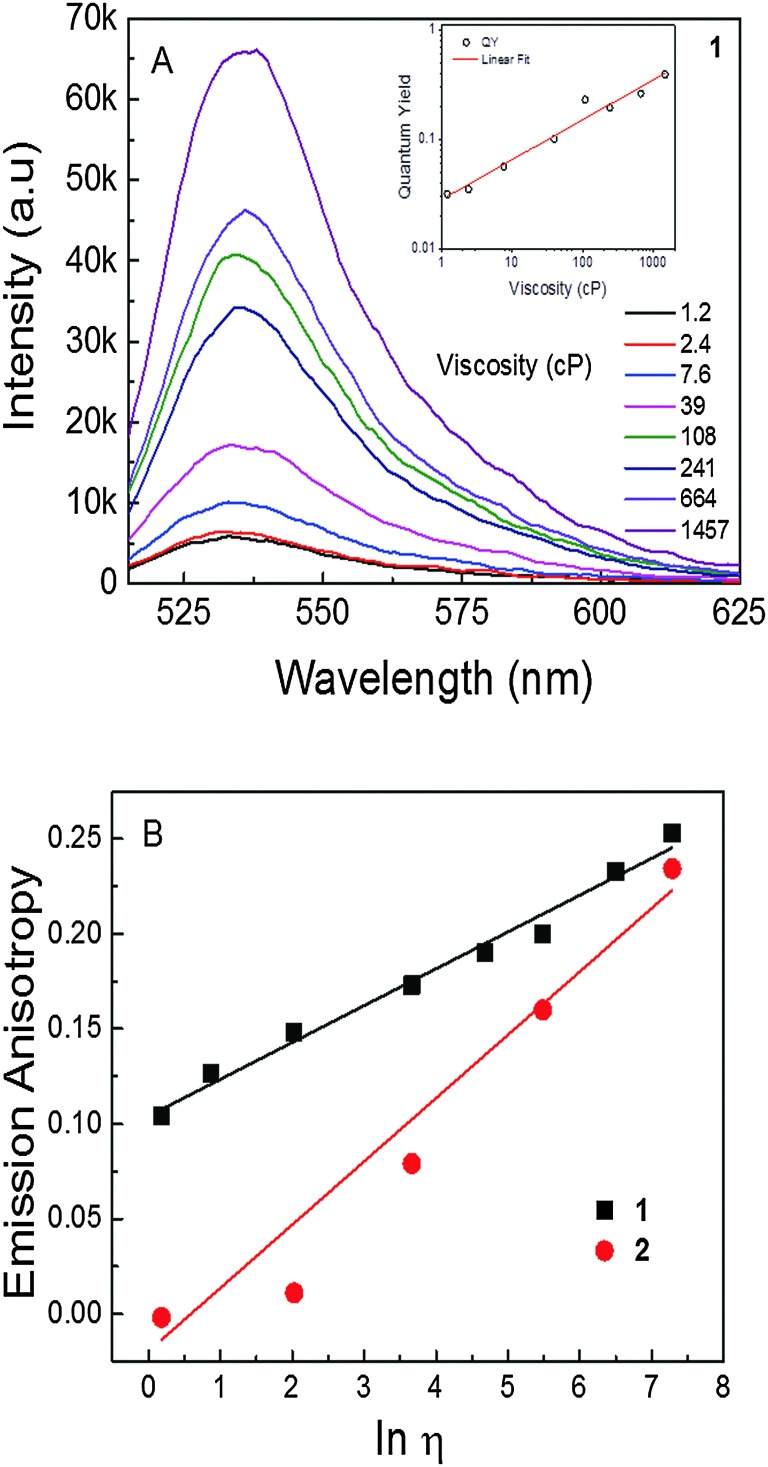
(A) Emission spectra of dye **1** in different viscosity mixtures of ethanol : glycerol; inset: quantum yield as function of viscosity. (B) Steady state emission anisotropy of **1** and **2** as a function of viscosity.

Furthermore, we examined how the emission of **1** and **2** depended on the viscosity of the media (Fig. 2A). Solvent mixtures with different viscosities were prepared by mixing the appropriate amounts of ethanol and glycerol. Emission intensity increased *ca.* 12 times as viscosity increased from 1.2 cP (ethanol) to 1457 cP (glycerol) in a linear manner, as expected for any molecular rotor based on the Förster–Hoffman theory. In addition, quantum yield was changed by *ca.* 12 fold (Fig. 2, inset). A less than 2-fold change in emission intensity was observed for non-rotor **2** in the same range of viscosities, while the quantum yield (0.60) remained unchanged (data not shown). Moreover, steady state emission anisotropy was also evaluated as a function of medium viscosity for both **1** and **2**. Emission anisotropy of 1 in ethanol was found to be around 0.1 and in glycerol it increased to 0.25. On the other hand, the anisotropy of **2**, *i.e.*, the non-rotor dye, in ethanol was found to be close to zero, yet in glycerol, the anisotropy was found to be *ca.* 0.25, *i.e.*, similar to that observed for rotor **1** in a solvent of high viscosity. The large difference in steady-state anisotropy in ethanol for both dyes was due to the difference in their respective fluorescence lifetimes. In ethanol, the lifetime of **1** (*i.e.*, rotor) was short (*ca.* 180 ps), which provided less time for the molecules in the excited state to depolarize the emission *via* Brownian rotation, thus leading to a relatively higher anisotropy. While in the case of **2** (*i.e.*, non-rotor), the lifetime in ethanol was longer (*ca.* 3.4 ns); within this time frame, the fluorescence emission was completely randomized, which resulted in a close to zero anisotropy. Steady-state anisotropy could be used in the cellular imaging of viscosity by splitting the emission signal into two different detectors and thus detecting vertical and perpendicular emission polarization components. This information was used to calculate the steady state anisotropy across the intensity image (Fig. 2B as an example of the calibration curve for determining viscosity).

**Fig. 3:**
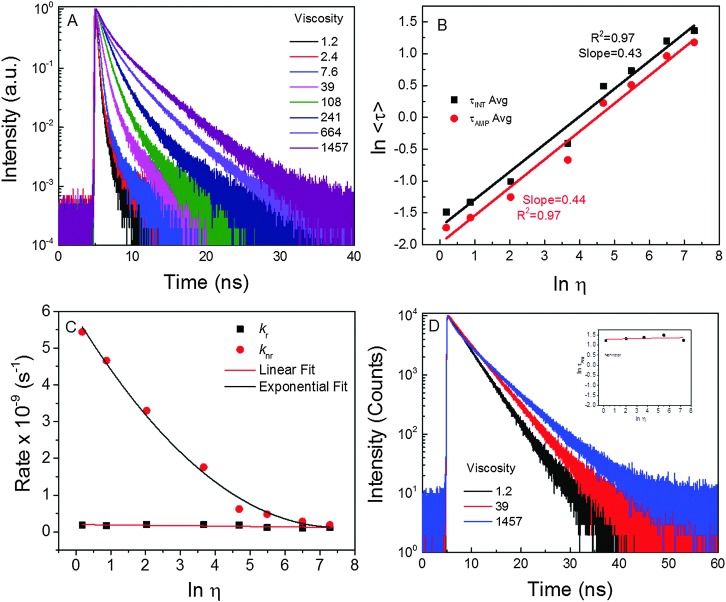
(A) Fluorescence intensity decays of rotor **1** in media of different viscosities (cP). (B) Average fluorescence lifetimes of **1** as function of viscosity. Fluorescence intensity decays of non-rotor in different viscosities; (C) radiative (kr) and non-radiative (knr) rates of **1** as a function of viscosity. (D) Intensity decays of **2** in media of different viscosity. Inset: Lifetime as a function of viscosity.

As previously mentioned, quantum yield and intensity are not reliable parameters for measuring viscosity due to their dependence on the local dye concentration, for example. Thus, we examined the fluorescence lifetimes of the dye in different viscosity media. Based on the fluorescence intensity decays of **1** in the solutions of different viscosities, an obvious change in the slope of decays with an increase of solvent viscosities was noted. Evaluating the fluorescence lifetime as a function of viscosity (Fig. 3B) suggested that the lifetime of rotor 1 changed *ca.* 18-fold (from 180 ps to 3245 ps) as the viscosity of media changed from 1.2 cP to 1457 cP. The slope of the linear fit for the rotor's data (Fig. 3B) was found to be 0.43 (which is close to the value of 0.6 for a perfect rotor). Typical slope values range from 0.2 to 1.4 for different types of rotors.^50^ Moreover, it is important to note that the radiative (*k*_r_) rate barely changed (Fig. 3C), while the non-radiative rate (*k*_nr_) changed by *ca.* 21 times as media's viscosity changed from 1.2 cP to 1457 cP. As expected, fluorescence intensity decays of dye **2** were virtually independent of viscosity (Fig. 3D; only 3 traces are shown due to strong overlap of the intermediate decays). The fluorescence lifetime only changed by about 1.5 times (from 3.44 ns to 4.97 ns) over the range of viscosities.

**Fig. 4:**
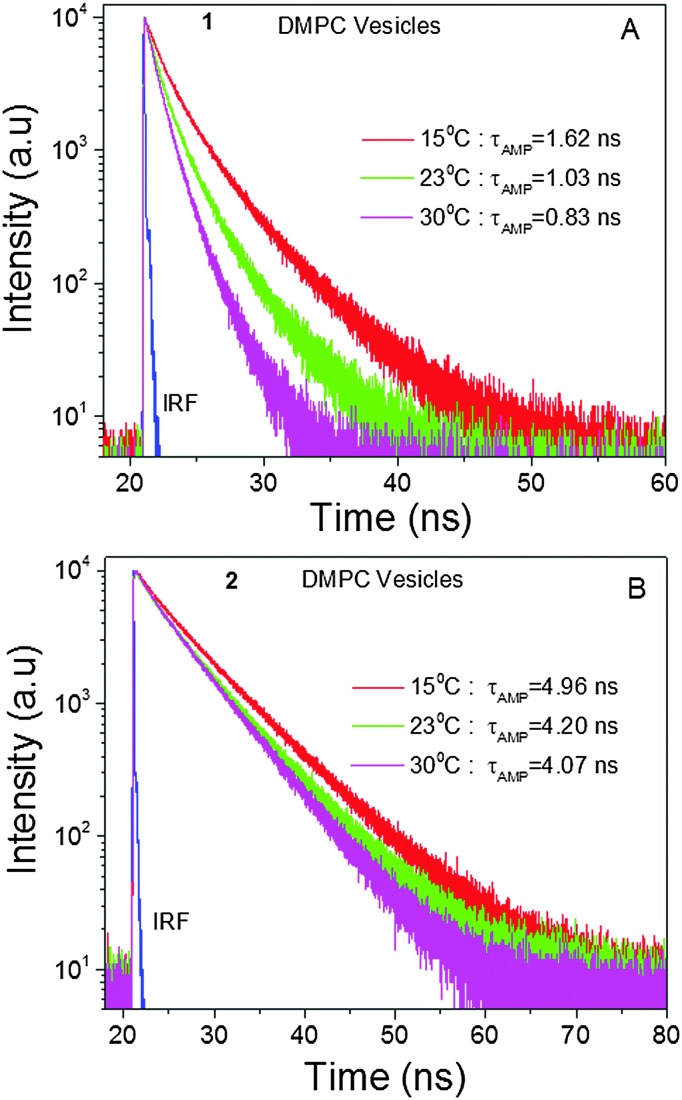
Intensity decays of **1** (A) and **2** (B) in DMPC vesicles as a function of temperature.

Next, in order to estimate the viscosity of membrane-like environments using BODIPY trimers, we encapsulated the trimer BODIPYs **1** and **2** into DMPC lipid vesicles and measured their fluorescence lifetimes at various temperatures (15, 23 and 30 °C). It is expected that at 15 °C, a gel state would be the dominant form, where the lipid molecules were rigidly packed. Thus, the lateral diffusion of lipid molecules as well as bond rotations should be suppressed. In contrast, at 30 °C a less viscous liquid state would be achieved, where the hydrocarbon chains were expected to be loosely arranged, thus the bond rotations should be allowed. Based on our data (Fig. 4) a 1.2-fold change in the fluorescence lifetime in the case of **2** could be ascribed to the temperature effect, since the non-rotor molecule is insensitive to viscosity changes that could be induced by temperature. However, the change in the case of the rotor molecule **1** was almost twice owing to changes in the surrounding viscosity along with the temperature effect. The measured viscosities of the DMPC vesicles using **1** were 270, 98 and 60 cP at 15, 23 and 30 °C, respectively. Arguably, the viscosity measurements (Fig. 4A) might slightly underestimate the viscosity in lipid bilayer as temperature, which could be viewed as an additional non-radiative channel. This would lower the lifetime values as compared to those expected from the viscosity effect alone. Nonetheless, it is plausible that **1** could be used as a molecular viscometer to estimate the viscosity in membrane mimicking vesicles and plasma membranes of various cells. Further, we considered how the polarity of the environment could affect the behaviour of the molecular rotor. Based on the photo-physical properties of **1** in organic solvents of different polarities, minimal changes in absorption, fluorescence emission spectra and lifetime (as the most important parameter) were observed (Fig. S1 and Tables S2, S3, ESI†). These experiments proved that the rotor's lifetime measurements for viscosity determination would not be affected by polarity variations of the media.

**Fig. 5:**
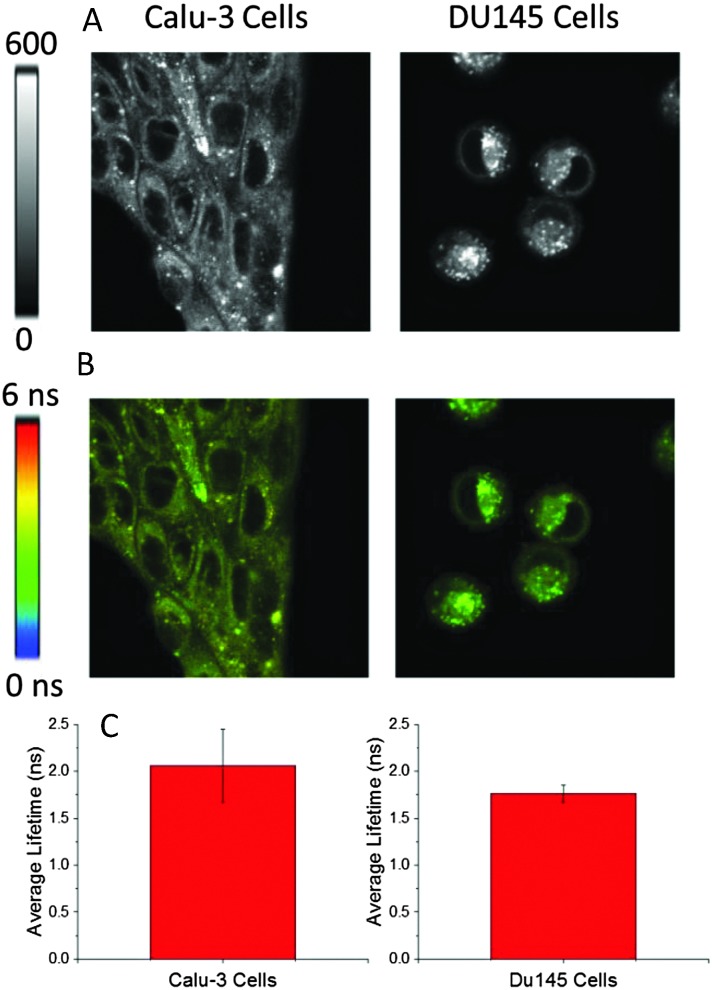
(A) 80 × 80 μm Intensity images of Calu3 and DU145 cells treated with 500 nM of **1** (rotor). (B) 80 × 80 μm FLIM images of Calu3 and DU145 cells treated with 500 nM of **1** (rotor). (C) Amplitude average lifetime recovered from FLIM images from respective cell lines (*n* = 6 images).

Further, we investigated the cellular distribution of **1** with the aim of measuring the intracellular viscosity. Towards this end, the behaviour of rotor **1** was tested in two different cancer cell lines (Fig. 5): Calu 3 (adenocarcinoma of the lung) and DU145 (a prostate cancer). It appeared that the distribution was bimodal in both cancer cell lines: diffuse fluorescence in cytoplasm and bright punctate distribution. Based on FLIM images (Fig. 5B) the fluorescence lifetime appeared to be long in both areas. Previously, we and others have observed this type of punctate behaviour which was attributed to the probe's accumulation in membranes of vesicle-like structures (mitochondria, lysosomes, endoplasmic reticulum and golgi apparatus *etc.*).^17,30^ A long lifetime and bright fluorescence in these punctate areas were expected due to a hydrophobic nature of the dyes as well as due to the viscosity experienced by the dye which was high compared to the cytoplasm/aqueous compartment. However, long lifetimes (which were comparable to the ones observed in punctate areas) in cytoplasm or throughout cells (Fig. 5B) were unusual (Fig. S2 and S3, ESI,† shows the lifetime profile along the line drawn in FLIM images of cell lines showing similar lifetimes in punctate area and cytoplasm). Potentially, this observation could be explained by the binding/physical adsorption of **1** to the cytoplasmic proteins or other cell components distributed throughout cytoplasm, which would increase the intensity and the fluorescence lifetime of the probe hindering the rotation around the central bond. Cell cytoplasm has a high concentration of cellular proteins which provide hydrophobic pockets for binding hydrophobic dyes. This hypothesis was tested by studying the binding of rotor **1** to several proteins *in vitro.* Based on the changes of the fluorescence lifetime, we found that rotor molecules interacted with larger proteins such as bovine serum and human serum albumins, but not with smaller proteins such as lysozyme (Fig. S4, ESI†). This information could be crucial in designing viscosity experiments in biologically relevant types of media.

## Conclusions

In summary, we showed that a triazine-based BODIPY trimer is a high molar extinction fluorophore, which is able to sense viscosity changes in various environments, including molecular solvents, lipid vesicles and several cancer cell lines. However, the analysis of the data in cellular environments should be done with care, since the changes in the fluorescence lifetimes (and subsequently the viscosity estimations) could be affected by the trimer binding to some proteins. This problem could potentially be solved by designing a fluorophore, which will specifically bind to a particular cell organelle or a compartment. These studies are currently underway in our laboratories.

In addition, the fluorescence properties of trimer **2** indicated that this dye could potentially be a good fluorophore in the green region with a high molar absorption coefficient, a high quantum yield (0.60) and a lifetime comparable to that of rhodamine dyes (*ca.* 4 ns). This further supports the notion that triazine could be a valuable scaffold for assembling multichromophoric systems.
