# Supplementary material for: A triazine-based BODIPY trimer as a molecular viscometer
Source: Phys Chem Chem Phys. 2016 Jan 21;18(6):4535–40. doi: 10.1039/c5cp07214j (PMC4755388; doi:10.1039/c5cp07214j)
Supplement: Supplementary file 1 [file CP-018-C5CP07214J-s001.pdf]

Electronic Supplementary Material (ESI) for Physical Chemistry Chemical Physics.
This journal is © the Owner Societies 2016

SUPPORTING INFORMATION

Triazene-based BODIPY trimer as a molecular viscometer

Sangram L. Raut,[a] Joseph D. Kimball,[a] Rafal Fudala,[b] Ilkay Bora,[ac†] Rahul Chib,[b] Hana Jaffari,[a] Marlius K. Castillo,[c] Nicholas W. Smith,[c] Sergei V. Dzyuba,*[c] and Zygmunt Gryczynski*[a]

[a] *Department of Physics and Astronomy, Texas Christian University, Fort Worth, TX 76129, USA. Fax: +1 817 257 7742; Tel: +1 817 257 4209; E-mail: z.gryczynski@tcu.edu*

[b] *Department of Cell Biology and Immunology, University of North Texas Health Science Center, Fort Worth, TX, 76129 USA*

[c] *Department of Chemistry and Biochemistry, Texas Christian University, Fort Worth, TX 76129, USA. Fax: +1 817 257 5851; Tel: +1 817 257 6218; E-mail: s.dzyuba@tcu.edu*

† *Visiting graduate student from the Department of Chemistry, University of Copenhagen, Universitetsparken 5, 2100 Copenhagen, Denmark*

TABLE OF CONTENTS

Materials and methods……………………………………………..…..……………………. 2

Synthesis and characterization of BODIPY dyes…..……………………………………. 2

Spectroscopic measurements…………………………………………………………… 4

Preparation of lipid vesicles……………………………………………………………… 5

Fluorescent microscopy and FLIM……………………………………………………… 5

Table S1 Extinction coefficient of BODIPY trimers in various solvents…... 7

Table S2 Lifetime parameters for dye **1** in organic solvents of different polarities…... 7

Table S3 Lifetime parameters for dye **1** in organic solvents of different polarities…… 8

Figure S1 Absorption and emission spectra of dye **1** (rotor; [**1**] = 0.5 μM) and dye **2** (non-rotor; [**2**] = 0.5 μM) in organic solvents of different polarity ……........…………………………………………………………………………….. 9

Figure S2 FLIM Image and lifetime Profile Calu3 Cells………………………………… 10

Figure S3 FLIM Image and lifetime Profile DU145 Cells……………………………….. 10

Figure S4 Fluorescence intensity decays of rotor **1** ([**1**] = 0.5 μM) in the presence and absence of different proteins **(**[protein] = 40 μM)………………………….. 11

References……………………….…………………………………………………..…. 12

Materials and Methods

All chemicals and solvents were from commercial sources (Aldrich, Acros, TCI America), they were of highest grade possible, and were used as received. ^{1}H, ^{13}C, ^{11}B and ^{19}F NMR spectra were recorded on a Bruker (400 MHz) spectrometer; the chemical shifts are reported in ppm (δ) downfield from tetramethylsilane in $CDCl_3$.

Synthesis and Characterization of BODIPY Dyes

Synthesis of dyes **A** and **B** were accomplished according to published procedures[1] and exhibited spectral properties consistent with their structures.[1a] Mechanochemical preparations[1b] are given below as examples.

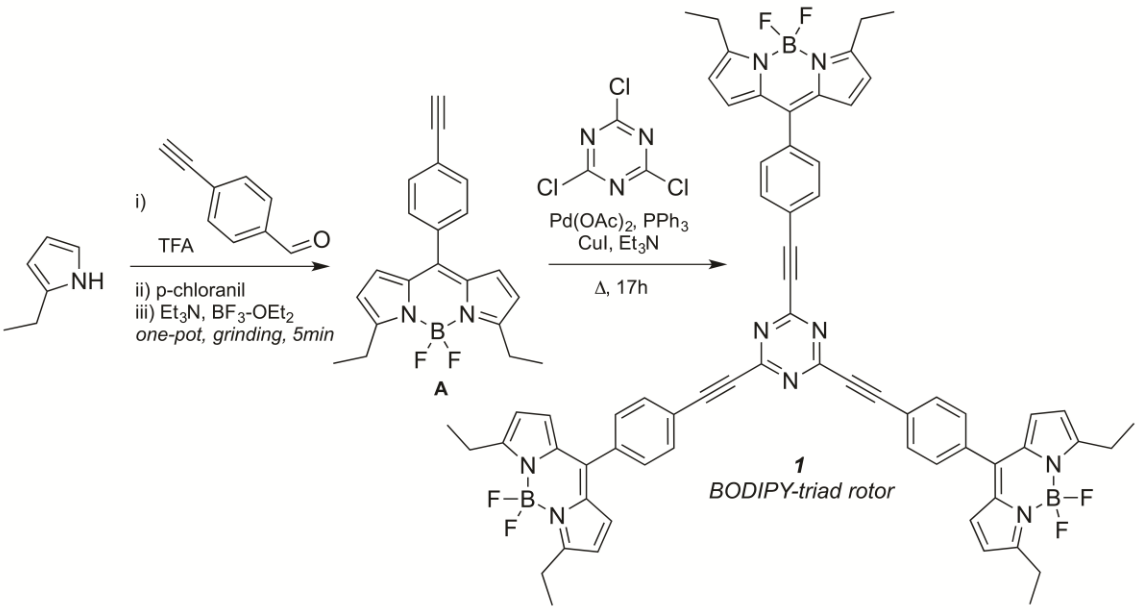

*Synthesis of dye **A**:*

In a hood behind the protecting shield, 2-ethylpyrrole (2.0 ml, 19.53 mmol) and 4-ethynylbenzaldehyde (1.14 g, 8.76 mmol) were mixed in a mortar with a pestle to form a suspension. Trifluoroacetic acid (TFA; 4-5 drops) was slowly added while grinding, which resulted in an almost instantaneous formation of a brown sticky paste. CH_2Cl_2 (*ca*. 2 ml) was added, followed by grinding to obtain homogeneous mixture. Next, p-chloranil (2.37g, 9.64 mmol) was added and grinded followed until deep red paste was obtained. Subsequently, Et_3N (10 ml, 71.65 mmol) was added, until the color of the mixture turned into a green/brown paste. Next, BF_3-OEt_2 (10 ml, 81 mmol) was added and grinded until a red metallic paste was formed. The resulting mixture was transferred into the separatory funnel with 400 ml of CH_2Cl_2 and carefully (without vigorous shaking, to avoid producing stable emulsion) washed with saturated K_2CO_3 solution (200 ml x 2) followed by brine (200 ml x 2). Volatiles were removed *in vacuo*, and the residue was purified by column chromatography (SiO_2/$CHCl_3$) to yield dye **A** (0.328 g, 10.75%) as a red solid.

^{1}H NMR (400 MHz, $CDCl_3$) δ 7.60 (d, *J* = 8.4 Hz, 2H), 7.46 (d, *J* = 8.4 Hz, 2H), 6.71 (d, *J* = 4.2 Hz, 2H), 6.36 (d, *J* = 4.2 Hz, 2H), 3.21 (s, 1H), 3.08 (q, *J* = 7.6 Hz, 4H), 1.34 (t, *J* = 7.6 Hz, 6H).

^{13}C NMR (100MHz, $CDCl_3$) δ 164.2, 141.9, 134.8, 134.2, 132.1, 130.6, 130.5, 124.1, 117.8, 83.0, 79.5, 22.3, 13.0.

^{19}F NMR (376 MHz, $CDCl_3$) δ – 145.22 (q, *J* = 34 Hz).

^{11}B NMR (128 MHz, $CDCl_3$) δ 0.97 (t, *J* = 34 Hz).

*Synthesis of dye **1**:*

A screwcap vial containing a stirring bar was subsequently charged with alkyne BODIPY **A** (4.5 eq, 20 mg, 57 μmol), cyanuric chloride (2.3 mg, 12.7 μmol), CuI (4.8 mg, 1.28 μmol), $Pd(OAc)_2$ (1.2 mg, 1.28 μmol), PPh_3 (2.0 mg, 6.38 μmol), and Et_3N (0.2 ml), capped and stirred under reflux for 17 hours. After cooling to room temperature, the mixture was quenched with HCl (1M, 20 ml) and extracted with CH_2Cl_2 (20 ml x 2). Organic fractions were combined, rotovaped, and the crude material was purified by column chromatography ($CHCl_3$/hexanes – 3/1 (v/v)) to obtain the triad **1** (0.0076 g, 26.6 % yield) as a red solid.

^{1}H NMR (400 MHz, $CDCl_3$) δ 7.66 (d, *J* = 8.5 Hz, 6H), 7.50 (d, *J* = 8.4 Hz, 6H), 6.73 (d, *J* = 4.2 Hz, 6H), 6.37 (d, *J* = 4.2 Hz, 6H), 3.09 (q, *J* = 7.6 Hz, 12H), 1.35 (t, *J* = 7.6 Hz, 18H).

^{13}C NMR (100 MHz, $CDCl_3$) δ 164.2, 141.5, 135.4, 134.1, 132.4, 130.7, 130.33, 123.5, 117.7, 81.7, 77.4, 75.8, 22.2, 12.9.

^{19}F NMR (376 MHz, $CDCl_3$) δ –145.23 (q, *J* = 33 Hz).

^{11}B NMR (128 MHz, $CDCl_3$) δ 0.97 (t, *J* = 33 Hz).

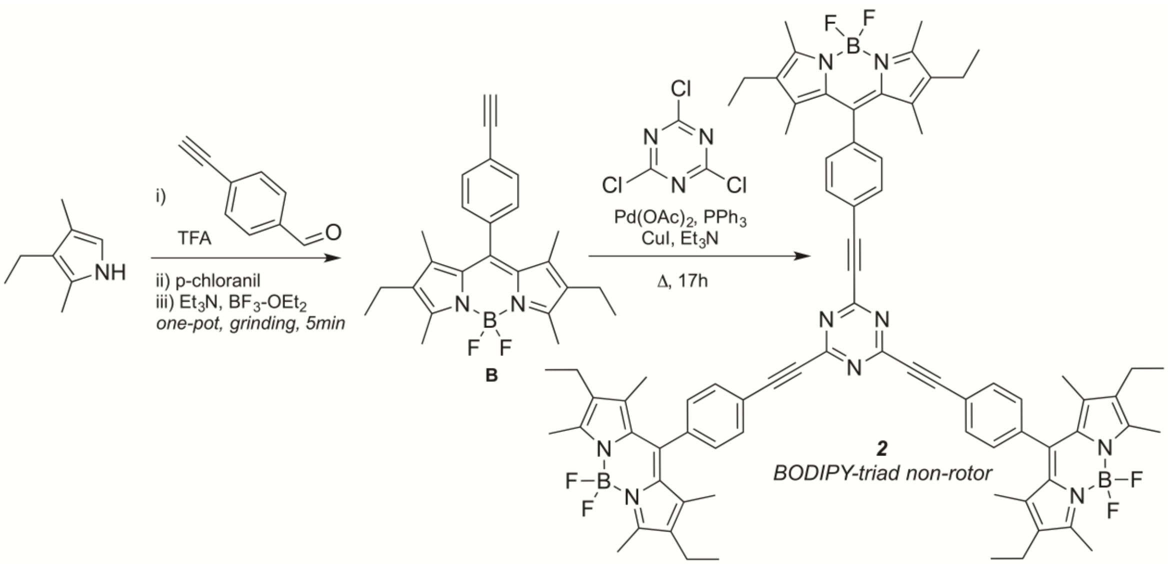

*Synthesis of dye **B**:*

In a hood behind a protective shield, 3-ethyl-2,4-dimethyl-1H-pyrrole (2.2 ml, 16.23 mmol) and 4-ethynylbenzaldehyde (0.93 g, 7.13 mmol) were grinded with a pestle to obtain a

suspension. TFA (3-5 drops) was slowly added which results in an almost instantaneous formation of a brown sticky mixture. CH_2Cl_2 (*ca*. 2 ml) was added, followed by grinding to obtain homogeneous mixture. Next, p-chloranil (1.91 g, 7.76 mmol) was added while grinding to obtain a dark red paste. Subsequent addition of Et_3N (10 ml, 71.65 mmol) while grinding produced a green/brown mixture, to which BF_3-OEt_2 (10 ml, 81 mmol) was added dropwise under grinding to produce a metallic red paste. The mixture was transferred into a separatory funnel using CH_2Cl_2 (500 ml) and carefully (without vigorous shaking, which produces stable emulsion) washed with saturated K_2CO_3 solution (200 ml x 2), followed by brined (200 ml x 2). Volatiles were removed *in vacuo* and the residue was purified by column chromatography (silica gel / $CHCl_3$) to obtain dye **B** (0.885 g, 31%) as a dark red solid.

^{1}H NMR (400 MHz, $CDCl_3$): δ 7.62 (d, *J* = 8.3 Hz, 2H), 7.27 (d, *J* = 8.4 Hz, 2H), 3.18 (s, 1H), 2.53 (s, 6H), 2.30 (q, *J* = 7.6 Hz, 4H), 1.30 (s, 6H), 0.98 (t, *J* = 7.6 Hz).

^{13}C NMR (100 MHz, $CDCl_3$): δ 154.3, 139.2, 138.4, 136.7, 133.2, 133.0, 130.7, 128.7, 122.9, 83.3, 78.7, 17.3, 14.8, 12.8, 12.1.

^{19}F NMR (376 MHz, $CDCl_3$): δ –145.8 (q, *J* = 33 Hz).

^{11}B NMR (128 MHz, $CDCl_3$): δ 0.78 (t, *J* = 33 Hz).

*Synthesis of dye **2**:*

A screwcap vial was sequentially charged with a stirring bar, alkyne BODIPY **B** (50 mg, 124 μmol), Et_3N (0.25 ml) cyanuric chloride (5.0 mg, 27 μmol), CuI (1.6 mg, 8.2 μmol), $Pd(OAc)_2$ (1.8 mg, 8.2 μmol) and PPh_3 (4.3 mg, 16.5 μmol). The vial was capped and stirred under reflux for 17 h. After cooling to room temperature, the mixture was diluted with CH_2Cl_2 (20 ml), washed with HCl (1M, 20 ml x 2), and the volatiles removed *in vacuo*. The residue was subjected to column chromatography (silica gel, $CHCl_3$) to obtain triad **2** (4.6 mg, 13 % yield) as a red solid.

^{1}H NMR (400 MHz, $CDCl_3$) δ 7.67 (d, *J* = 8.5 Hz, 6H), 7.31 (d, *J* = 8.5 Hz, 6H), 2.54 (s, 6H), 2.31 (q, *J* = 7.5 Hz, 12H), 1.32 (s, 18H), 0.99 (t, *J* = 7.5 Hz, 18H).

^{13}C NMR (100 MHz, $CDCl_3$) δ 154.4, 138.9, 138.2, 137.2, 133.3, 133.2, 130.6, 128.9, 122.4, 81.7, 77.4, 75.0, 17.2, 14.8, 12.7, 12.1.

^{19}F NMR (376 MHz, $CDCl_3$) δ –145.8 (q, *J* = 33 Hz).

^{11}B NMR (128 MHz, $CDCl_3$) δ 0.78 (t, *J* = 33 Hz).

Spectroscopic Measurements

UV-Vis absorption and fluorescence spectra were obtained using a Cary 50 bio UV–visible spectrophotometer (Varian) and Cary Eclipse spectrofluorometer (Varian), respectively. All measurements were conducted using quartz 0.4 x 1cm cuvettes at room temperature with optical density below 0.05, unless mentioned otherwise. In order to measure the quantum yield, absorption spectra of the BODIPY trimers were collected followed by measuring the integrated fluorescence intensity of the sample. A solution of rhodamine B in ethanol was used as a reference (quantum yield: 0.7).[2]

Steady state anisotropy was measured by observing the emission from sample in vertical (parallel) and horizontal (perpendicular) emission polarizer orientation with respect to excitation

polarizer. The obtained emission data were used to calculate the steady state emission anisotropy (r) using the following formula:

$$r = \frac{I_{II} - GI_{\perp}}{I_{II} + 2 * GI_{\perp}} \qquad (1)$$

where, G is the instrumental correction factor, I_{II} is the intensity in parallel configuration of excitation and emission polarizer and $I_{\perp}$ is the intensity in perpendicular configuration of excitation and emission polarizer. Fluorescence lifetime was measured on a FluoTime 300 fluorometer (PicoQuant, Inc.) Laser excitation was provided by a Supercontinuum WhiteLase SC-400 (Fianium, Ltd) pulsed white light laser which was passed through a hybrid quartz prism and grading monochromator to choose an excitation light of 500±10 with resolution set to 4 ps/channel. The fluorometer was equipped with an ultrafast microchannel plate MCPPMT detector from Hamamatsu. The fluorescence lifetimes were measured in the magic angle condition (54.70) and data analyzed using FluoFit4 program from PicoQuant, Inc (Germany) using multi-exponential fitting model:

$$I(t) = \sum_i \alpha_i e^{-t/\tau_i} \qquad (2)$$

where, αi is the amplitude of the decay of the ith component at time t and τ_i is the lifetime of the ith component. The intensity weighted average lifetime (τ_{avg}) was calculated using following equation:

$$\tau_{avg} = \sum_i f_i \tau_i \quad \text{where} \quad f_i = \frac{\alpha_i \tau_i}{\sum_i \alpha_i \tau_i} \qquad (3)$$

Relative radiative and non-radiative rates were calculated using experimentally measured quantum yield and fluorescence lifetimes according to the following equation:

$$\phi_i = \frac{k_r}{k_r + k_{nr}} \quad \text{and} \quad \tau = \frac{1}{k_r + k_{nr}} \qquad (4)$$

Preparation of Lipid Vesicles

Lipid unilamellar vesicles were prepared using 1,2-dimyristoyl-sn-glycero-3-phosphocholine (DMPC). Briefly, appropriate amount of lipid stock (725 µL of 1.4 mM $CHCl_3$ stock) and BODIPY trimers (10 µL of 100 µM DMSO stock) were mixed (lipid:dye ratio was *ca*. 1000:1) in glass bottles. The solvents were evaporated under moisture -free nitrogen stream and left overnight to remove any traces of organic solvents. Next, 1 mL of PBS buffer (pH 7.4) was added, followed by sonication at about 40 ^{o}C for 10 min to obtain multilamellar vesicles. In order to obtain unilamellar vesicles, these mutlilamellar vesicles were passed through 100 µm and 0.02 µm membrane filters attached to syringe filter in a cascade manner once to obtain unilamellar vesicles.

Fluorescence Microscopy and FLIM

Calu 3 (human epithelial lung cancer cells) and DU145 (human epithelial prostate cancer cells) cancer cell lines were obtained from the American Type Culture Collection (ATCC), Manassas, VA (USA) and were grown to 70 % confluence in RPMI supplemented with 10% FBS and 1% Pen-Strep. Cells were trypsinized using 0.25 % Trypsin EDTA and seeded on 20 mm round glass-bottom petri dishes. After 24 hours, the cells were stained with 500 nM solution of BODIPY trimer in DMSO for 20 min at 37 ^{o}C (10 µL DMSO in 1 mL of cell media). Next, the

media was washed 3 times using PBS and fresh PBS was added followed by FLIM imaging on Olympus IX7 microscope. Laser excitation was provided by a pulsed laser diode (PDL-470) emitting 470 nm light and driven by a PDL 828 "Sepia II" driver (operated at 20 MHz). Measurements were performed on a MicroTime 200 time-resolved, confocal microscope (PicoQuant). The excitation and emission light was focused by a 60X 1.2 NA Olympus objective in an Olympus IX71 microscope, and the emission light was filtered by a 488 long wave pass filter before passing through a 50 µm pinhole. The detection was achieved by a hybrid photomultiplier assembly. The resolution of the time correlated single photon counting (TCSPC) module was set to 4 ps/bin in order to facilitate the detection at highest possible resolution. All data analysis were performed using the SymPhoTime software, version 5.3.2. All experimental equipment and the SymPhoTime software were provided by PicoQuant, GmbH as part of the MicroTime 200 system.

Table S1. Extinction coefficient (λ^{abs}_{max}) of dyes **1** and **2** in various solvents

**SOLVENT**	**ε / M^{-1} cm^{-1}**	
	**1**	**2**
ethanol	206000	235000
glycerol	99000	99000
ethanol/glycerol – 90/10 (v/v)	189000	232000
ethanol/glycerol – 50/50 (v/v)	193000	325000
ethanol/glycerol – 10/90 (v/v)	92000	75000
1,2-dichloroethane	174000	242000
dimethylsulfoxide	150000	216000
PBS buffer, pH 7	62000	99000

Table S2: Lifetime parameters for dye **1** in organic solvents of different polarities

**solvents**	**τ1**	**τ2**	**τ3**	**α1**	**α2**	**α3**	**τ_{ave} INT**	**τ_{ave} AMP**	**χ^2**
1,4-dioxane	0.22	0.52	2.10	0.88	0.11	0.004	0.35	0.27	0.82
2-propanol	0.19	0.58	1.82	0.93	0.06	0.010	0.38	0.24	0.80
Chloroform	0.24	0.51	2.23	0.88	0.11	0.010	0.35	0.27	0.80
Ethanol	0.16	0.62	1.92	0.95	0.04	0.010	0.31	0.19	0.80
Toluene	0.26	0.58	2.16	0.89	0.10	0.010	0.38	0.30	0.80
Dichloromethane	0.18	0.45	2.30	0.90	0.10	0.003	0.30	0.21	0.78
Acetone	0.12	0.48	2.40	0.97	0.03	0.005	0.23	0.14	0.80
Dimethylsulfoxide	0.18	0.50	1.70	0.88	0.11	0.004	0.34	0.23	0.81

Table S3: Lifetime parameters for dye **2** in organic solvents of different polarities

solvents	$\tau 1$	$\tau 2$	$\tau 3$	$\alpha 1$	$\alpha 2$	$\alpha 3$	τ_{ave} INT	τ_{ave} AMP	χ^2
1,4-dioxane	0.24	3.3	4.0	0.04	0.28	0.68	3.8	3.70	0.96
2-propanol	0.25	2.0	3.7	0.05	0.11	0.84	3.6	3.40	0.95
Chloroform	0.19	2.4	3.8	0.03	0.12	0.85	3.7	3.50	0.98
Ethanol	0.26	2.1	3.9	0.06	0.10	0.84	3.8	3.50	1.00
Toluene	0.20	3.1	3.7	0.03	0.44	0.53	3.5	3.30	0.99
Dichloromethane	0.29	2.4	3.8	0.05	0.17	0.78	3.6	3.40	0.97
Acetone	0.20	2.5	3.5	0.06	0.35	0.59	3.2	2.95	1.00
Dimethylsulfoxide	0.30	2.5	3.7	0.07	0.25	0.68	3.4	3.20	0.97

Figure S1: Absorption and emission spectra of dye **1** (rotor; [**1**] = 0.5 μM) and dye **2** (non-rotor; [**2**] = 0.5 μM) in organic solvents of different polarity

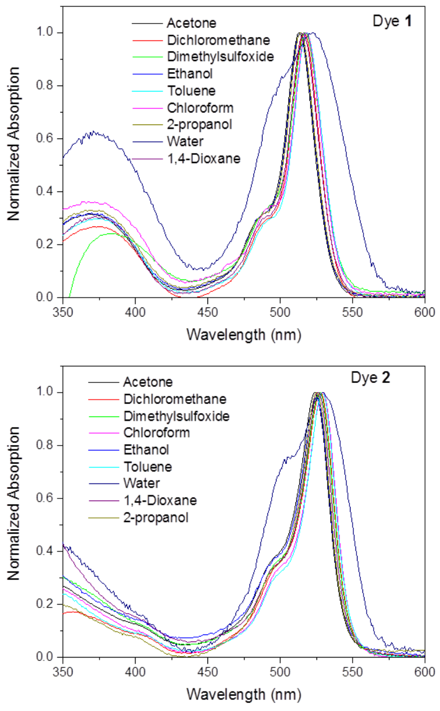

Figure S2: FLIM Image and lifetime Profile Calu3 Cells: FLIM image of Calu 3 cells along with lifetime profile along the red arrow drawn in the FLIM image. Red arrow was drawn such that it will pass through cytoplasm area and punctate area as well. Lifetime showed on the right as intensity weighted lifetime.

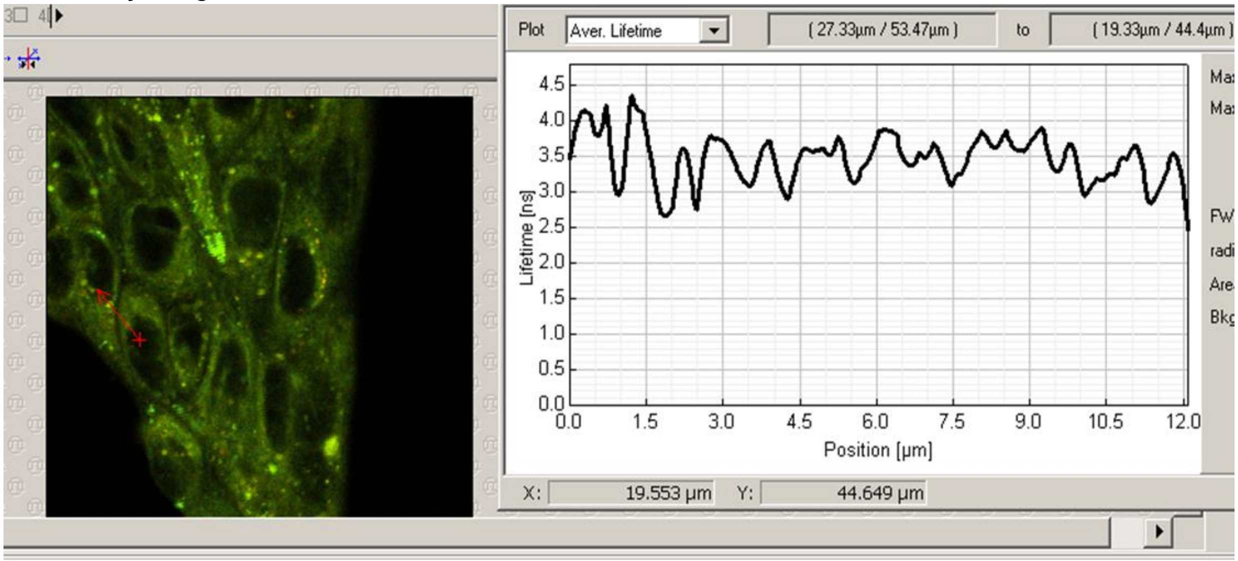

Figure S3: FLIM Image and lifetime Profile DU145 Cells: FLIM image of DU145 cells along with lifetime profile along the red arrow drawn in the FLIM image. Red arrow was drawn such that it will pass through cytoplasm and punctate area as well. Lifetime showed on the right as intensity weighted lifetime.

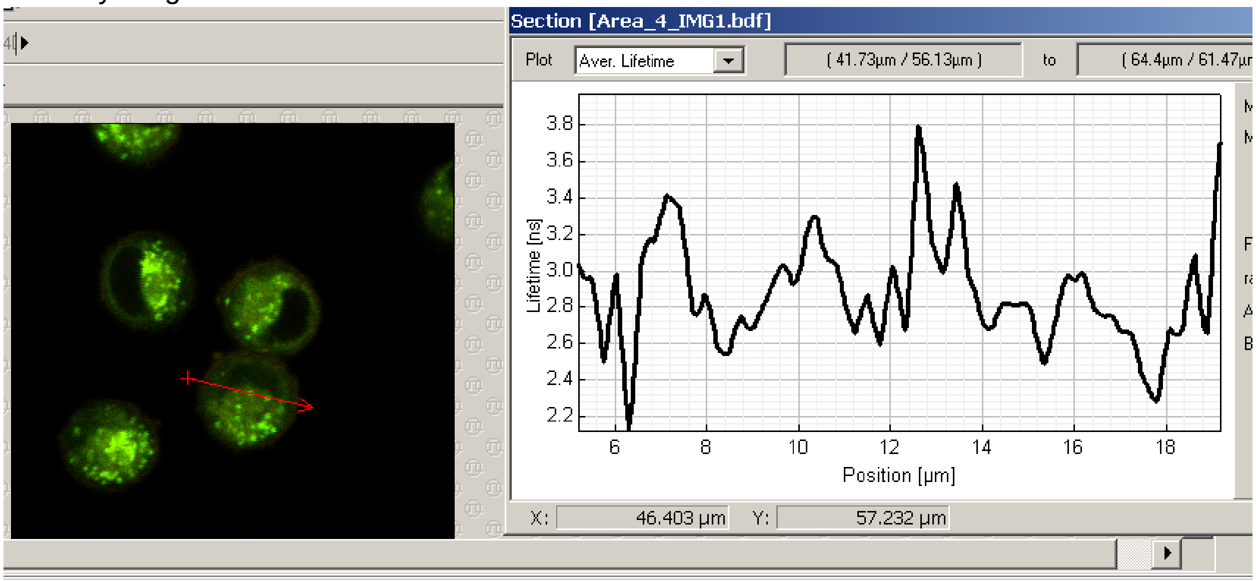

Figure S4: Fluorescence intensity decays of rotor **1** (**[1]** = 0.5 μM) in the presence and absence of different proteins **(**[protein] = 40 μM); bovine serum albumin (BSA) and human serum albumin (HSA).

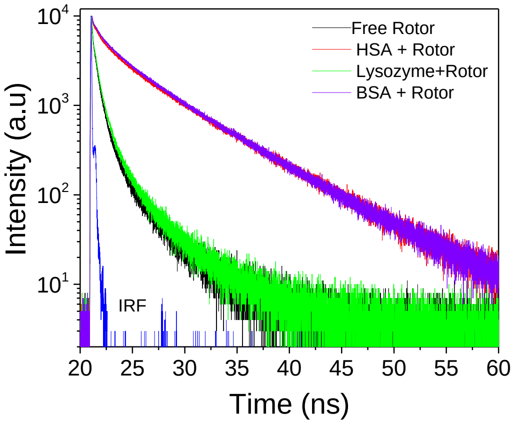

References

1. (a) N. W. Smith, A. Alonso, C. M. Brown and S. V. Dzyuba, *Biochem. Biophys. Res. Commun.,* 2010, **391**, 1455–1458; (b) L. P. Jameson and S. V. Dzyuba, *Beilstein J. Org. Chem.,* 2013, **9**, 786–790.
2. F. L. Arbeloa, P. R. Ojeda and I. L. Arbeloa, *J. Lumin.,* 1989, **44**, 105–112.
